# Broken/dislodged mediport catheter in the right heart

**DOI:** 10.11604/pamj.2023.44.80.39196

**Published:** 2023-02-09

**Authors:** Siva Naga Yarrarapu, Pratik Panchal

**Affiliations:** 1 Department of Internal Medicine, Monmouth Medical Center, RWJ Barnabas Health System, New Jersey, United States of America,; 2 Department of Cardiology, Monmouth Medical Center, RWJ Barnabas Health system, New Jersey, United States of America

**Keywords:** Dislodged, mediport, catheter

## Image in medicine

Spontaneous fragmentation and dislocation of Central venous assist devices (CVAD), like port-a-cath, is a rare complication with an incidence of 0.4% to 1.8%. Presentation can be asymptomatic or may have serious implications including arrhythmias, vessel rupture, thrombosis, pseudoaneurysm formation and perforation of the heart. Our case describes a 67-year-old female with history of signet cell adenocarcinoma of colon, who had a port-a-cath placed for chemotherapy about 16 months prior. Patient presented with problems with regards to her mediport including difficulties in flushing during the administration of chemotherapeutic agents. Chest X-ray was notable for broken/dislodged mediport catheter in the right heart. The patient was placed on cardiac monitoring and no arrhythmias were identified. The patient denied chest pain or shortness of breath. On evaluation, there was a lack of blood return from portacatheter despite recent attempts at declotting using tissue plasminogen activator (tPA). Contrast injection of the Central venous access device (CVAD) was done including fluoroscopy/imaging. The portacatheter was accessed with a Huber needle. Contrast injection revealed that the catheter had fragmented with contrast passing into the superior vena cava. An approximately 10 cm length catheter fragment was identified within the right atrium and right ventricle. Spontaneous fragmentation of portacatheter at the level of entry into the right subclavian vein was noted that migrated to the right heart. Retrieval of broken/dislodged mediport catheter from the right heart was done via right femoral vein access. She was transported on a cardiac monitor out of the department for admission to telemetry in stable condition.

**Figure 1:**
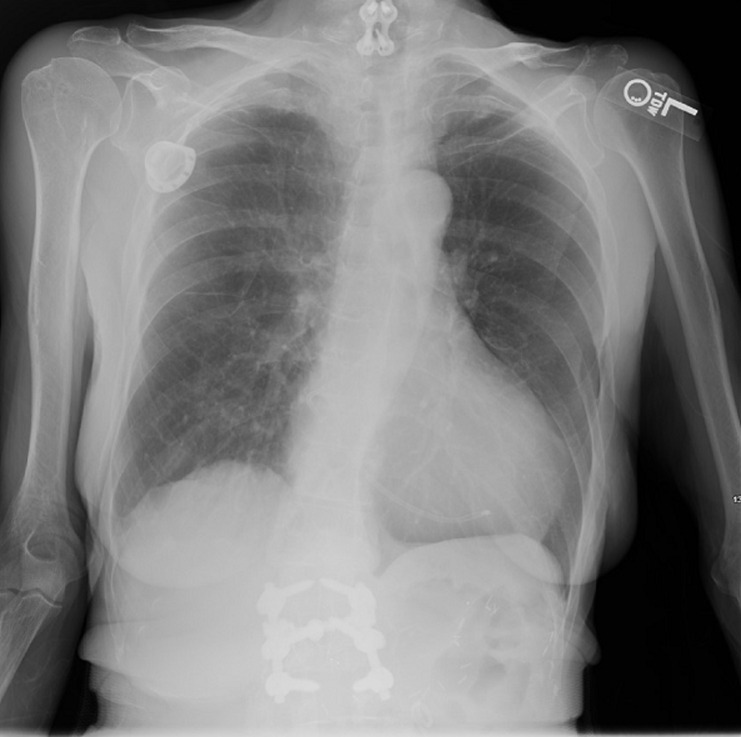
broken/dislodged mediport catheter in the right heart

